# Comments on “Use of hydrochlorothiazide and risk of skin cancer: a nationwide Taiwanese case-control study”

**DOI:** 10.1038/s41416-020-0791-0

**Published:** 2020-03-16

**Authors:** Erfan Taherifard

**Affiliations:** MPH and Medical student at Shiraz University of Medical Sciences, Fars Province, Shiraz, Zand Iran

**Keywords:** Cancer epidemiology, Oral cancer

## To the Editor,

I recently read with great interest the article written by Dr. Pottegård et al. entitled “Use of hydrochlorothiazide and risk of skin cancer: a nationwide Taiwanese case-control study”, published on 17 September 2019.^1^ The study evaluated the relationship between hydrochlorothiazide application and risk of skin malignancies in an Asian population using a case-control design. This study concluded that the use of this agent is not associated with increased risk of skin cancers, including lip and non-lip non-melanoma skin cancers and melanomas. The study is impressive, and has been conducted with a good population size; however, there are some concerns that may endanger the validity of the study.

First, the potential for confounding in this study cannot be completely assessed; this issue is modifiable simply by stratification of the control column in Table 1 by exposure, which allows us as readers to compare the distribution of potential confounders between these two groups of the controls: exposed and unexposed.^2^ Furthermore, as one of the aims of this study was to evaluate whether the risks reported about hydrochlorothiazide use in Caucasian populations are generalisable to Asian populations or not, it would be more interesting if the authors had assigned a column to variable status in that population, allowing the readers to have a direct comparison between the two populations. Lastly, the article lacks information about missing data, how the authors approached this and what analytic option was applied for the handling.

I believe that addressing these issues in the article would help readers gain a better insight into the results of the research, and improve the quality of the work.

## Data Availability

Not applicable.
